# ECG Markers of Positive Drug Challenge With Ajmaline in Patients With Brugada Syndrome

**DOI:** 10.1111/anec.70137

**Published:** 2025-12-08

**Authors:** Erol Tülümen, Mathieu Kruska, Sara Wuerfel, Maximilian Kohl, Volker Liebe, Ibrahim Akin, Juergen Kuschyk, Daniel Duerschmied, Martin Borggrefe, Boris Rudic

**Affiliations:** ^1^ Department of Internal Medicine, Cardiology, Angiology, Haemostaseology, and Medical Intensive Care, Medical Centre Mannheim, Medical Faculty Mannheim Heidelberg University Mannheim Germany; ^2^ Koc University Hospital Istanbul Turkey

**Keywords:** ajmaline challenge, Brugada syndrome, ECG markers, provocation testing

## Abstract

**Background:**

Ajmaline challenge (AC) is used for diagnosing suspected Brugada syndrome (BS) in patients with unexplained syncope, survived cardiac arrest, or for family screening.

**Purpose:**

To evaluate baseline ECG markers predicting a positive AC in the absence of a spontaneous diagnostic Brugada ECG.

**Methods:**

Baseline ECGs of 221 consecutive patients undergoing AC (up to 1 mg/kg bodyweight) were analyzed. ECGs from positive and negative tests were compared, with Q‐, R‐, S‐, J‐, and T‐wave amplitudes and intervals measured in all 12 leads.

**Results:**

221 patients underwent AC; the cohort was 71% male, and 7% had survived cardiac arrest. AC was positive in 93 patients (42%). Prominent S‐waves in lead II and J‐waves in V1 predicted a positive AC (S‐wave duration: 36 vs. 22 ms, *p* < 0.01; J‐wave amplitude V1: 0.06 vs. 0.01 mV, *p* < 0.001). ROC analysis confirmed discriminative value for S‐wave duration in lead II (AUC 0.79) and J‐wave amplitude in V1 (AUC 0.71). A cut off of ≥ 19 ms for S‐wave duration in lead II showed 96% sensitivity for a positive test (OR 17.3, *p* < 0.001). J‐wave amplitude in V1 ≥ 0.05 mV was also significantly associated (OR 5.4, *p* < 0.001).

**Conclusion:**

In patients without a spontaneous diagnostic Brugada ECG, prominent S‐waves in lead II and J‐waves in V1 are subtle electrical abnormalities that help identify patients and family members with a higher likelihood of positive AC.

## Graphical abstract

A prominent S‐wave in lead II on a nondiagnostic baseline ECG is a powerful predictor for unmasking Brugada syndrome during an ajmaline challenge. This subtle sign enhances patient selection for provocation testing and aids in risk stratification, particularly in familial screening.

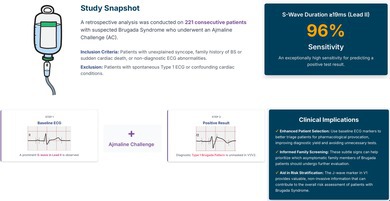

## 1 Introduction

Brugada syndrome (BS) is a primary electrical cardiac disorder associated with increased risk of spontaneous ventricular tachyarrhythmias and/or sudden cardiac death (SCD). The diagnosis is derived from typical coved‐type ST segment elevations in the right‐precordial leads V1 or V2 positioned in the second, third or fourth intercostal space either spontaneously or following administration of sodium channel blockers (Zeppenfeld et al. 2022). Up to 80% (Honarbakhsh et al. 2021) of all BS patients do not exhibit a spontaneous diagnostic type 1 ECG, but were diagnosed utilizing a sodium channel blocker test. Drug provocation testing with class IA (ajmaline) or class IC (flecainide) antiarrhythmic agents is recommended to unmask diagnostic ECG in patients suspected to have BS, including family members of definite BS cases. Several studies have compared the sensitivity and specificity of different provocation agents including ajmaline, flecainide, pilsicainamide, and procainamide (Wolpert et al. 2005; Somani et al. 2014). Although still no “gold standard” exists, current evidence suggests that ajmaline has the highest sensitivity and is thus preferable over flecainide (Therasse et al. 2017).

In previous studies, electrocardiographic markers of conduction anomalies within the right ventricular outflow tract (RVOT), commonly understood as augmented R‐wave in lead aVR, prominent S‐wave in leads I, II, and III, and larger S‐wave in lead II than in lead III (SII > SIII pattern) were shown to be more frequent in BS. Furthermore, S‐wave in lead I, SII > SIII pattern and prominent R‐wave in lead aVR were previously identified as an indicator of increased arrhythmic risk (Bayés de Luna and ABs 2012; Ragab et al. 2017, 2018; Calo et al. 2016). Whether Brugada patients with nondiagnostic ECG also bear subtle ECG changes reflecting RVOT conduction anomaly, and when present, the diagnostic and prognostic value of those changes are not known.

This retrospective analysis sought to describe baseline ECG characteristics associated with a positive ajmaline challenge (AC) aiding to improve the diagnostic yield of the BS.

## 2 Methods

### 2.1 Study Population

255 consecutive patients/probands with suspected BS underwent intravenous AC from 2015 to 2021. Indication for AC was based on family history of BS, family history of SCD, unexplained syncope, a basal nondiagnostic saddleback type ECG (i.e., Brugada pattern type 2) and survived unexplained cardiac arrest. Gross structural cardiac abnormalities were excluded in all patients. Comprehensive physical examination, medical history, transthoracic echocardiography, and bicycle ergometry were performed in all patients. 34 patients with negative AC and history of ventricular fibrillation (VF) were excluded from the analysis to prevent overlap with possible ECG abnormalities not related to BS. Patients with spontaneous type 1 ECG were excluded. In total, 221 patients with available follow‐up data were studied.

### 2.2 Ajmaline Challenge

**FIGURE 1:**
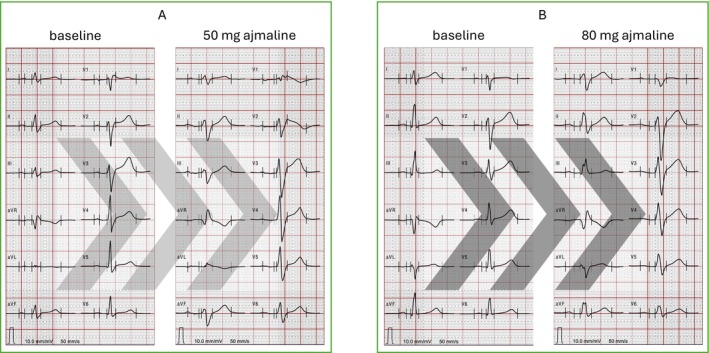
Example of positive (panel A) and negative (panel B) ajmaline challenge (AC) with a target dose of either 1 mg/kg or incremental administration until the first occurrence of the diagnostic Brugada ECG in at least one ECG lead.

After obtaining informed consent, ajmaline was administered intravenously over 5–10 min up to a maximum of 1 mg/kg body weight, following the recommended protocol of the Brugada consensus conferences published in 2005 and 2017 (Antzelevitch et al. 2017). Concomitant antiarrhythmic drugs or drugs known to provoke a Brugada ECG were discontinued for at least five half‐lives prior to AC. Infusion was discontinued when the diagnostic type 1 ECG pattern with ST segment elevation ≥ 0.2 mV with an inverted T‐wave appeared in at least one precordial lead positioned in the 2nd, 3rd, or 4th intercostal space (Zeppenfeld et al. 2022). Further criteria for discontinuing the testing were the occurrence of PVCs or VT, prolongation of the QRS duration to ≥ 130% compared to baseline or the occurrence of higher degree AV‐block. Figure 1 shows a representative example of a positive (1A) and negative (1B) response to AC.

### 2.3 ECG Analysis

In all patients, baseline ECGs immediately prior to the AC were automatically analyzed by CS‐200 Diagnostic System (SDS‐200 version 2.43 for Windows, Schiller AG, Baar, Switzerland). All measurements were manually and independently validated by MK and BR blinded to the outcome of the drug provocation test. In case of disagreement, a third electrophysiologist was consulted. Duration and amplitudes of the P wave, PQ, QRS, ST segment, and QT duration were measured in all ECG leads individually. Calipers were set to the earliest positive or negative deflection of the measured ECG component (P, Q, R, S, J, and T_end_) individually for every lead. The measurement of the QT interval was performed by Lepeschkin's method (“teach the tangent”); a tangential line was drawn from the peak of the T‐wave and the endpoint of the QT segment was derived from the intersection with the isoelectric line (Postema and Wilde 2014). ECGs were considered diagnostic (i.e., type 1) if the coved‐type pattern with ST segment elevation ≥ 0.2 mV was observed in any precordial lead (Zeppenfeld et al. 2022; Priori et al. 2015).

### 2.4 Statistics

Statistical analyses were conducted using IBM SPSS Statistics (version 27 for Mac, IBM Corporation, Somers, NY, USA), with descriptive statistics reported as median (interquartile range) and categorical variables as number and proportion. Testing for normal distribution was performed using the Shapiro–Wilk‐Test. Interval scale characteristics between the different groups were analyzed using the independent‐samples Mann–Whitney *U* Test. Chi square and Fisher's exact test were used for comparison of categorical variables. *p*‐values of < 0.05 were considered significant. Receiver‐operating‐characteristic (ROC) curves were calculated to identify the optimal discriminative cut off values for variables that differed among ajmaline‐positive and ajmaline‐negative patients. The Youden index was used to derive the optimal cut off value which was used in odds ratio (OR), sensitivity and specificity analyses. Univariate analysis was performed to individuate ECG predictors associated with positive ajmaline testing and occurrence of VF/SCD during follow‐up. To assess for multicollinearity between overlapping ECG parameters (e.g., QRS duration, S‐wave amplitude and S‐wave duration) prior to inclusion in the multivariable model, Pearsons's correlation coefficients were calculated. Multivariable analysis using logistic regression analysis was performed to individuate independent risk factors for positive AC and VF/SCD.

## 3 Results

### 3.1 Study Population

Demographic and clinical characteristics of the study population are summarized in Table 1. 221 patients underwent AC. 157 patients (71%) were male and median age was 37 [IQR 24]. The clinical context leading to AC was: survived cardiac arrest (7%), family history of BS or unexplained sudden cardiac arrest (31%) and at least one unexplained syncope with minor ECG abnormalities in the precordial leads (62%).

**TABLE 1:** Baseline demographics of the study population.

Total number of included patients, *N* (%)	221 (100%)
Age, years	37 [IQR 24]
Gender, male (%)	157 (71%)
Body‐mass‐index (kg/m^2^)	24.7 [IQR 5]
Positive response to ajmaline challenge, *N* (%)	93 (42%)
Indication for ajmaline challenge	
History of survived cardiac arrest, *N* (%)	15 (7%)
Family history of Brugada syndrome, *N* (%)	69 (31%)
ECG abnormalities and unexplained syncope, *N* (%)	137 (62%)
ECG parameters	
Heart rate baseline (bpm)	72 [IQR 14]
PR duration (ms)	162 [IQR 32]
QRS duration (ms)	94 [IQR 16]
QTc duration (ms)	411 [IQR 32]
First‐degree AV block, *N* (%)	24 (11%)
Type 2 Brugada ECG, *N* (%)	62 (28%)

### 3.2 Outcome and Side Effects of Ajmaline Challenge

AC resulted positive in 42% of patients (*n* = 93). In this group a diagnostic type 1 ECG was provoked in two ECG leads in 45 patients (48%) in three leads in 36 patients (39%) and in ≥ 4 ECG leads in 12 patients (13%). Ajmaline dose applied was significantly lower in ajmaline‐positive as compared to ajmaline‐negative patients (0.87 mg/kg body weight [IQR 0.31] vs. 1 mg/kg body weight [IQR 0.06]; *p* < 0.001). Three patients experienced adverse side effects during AC: one patient (ajmaline‐positive) developed VF that required defibrillation, one patient (ajmaline‐positive) had an onset of atrial fibrillation with spontaneous return to sinus rhythm and one patient (ajmaline‐negative) showed transient asystole due to sinoatrial block over 20 s with spontaneous recovery.

History of syncope was more frequent in patients with negative AC (54% vs. 32%, *p* = 0.002). Patients with positive AC were significantly older than patients with negative AC (42 years vs. 36 years, *p* = 0.007).

### 3.3 ECG Characteristics Associated With a Positive Ajmaline Challenge

**FIGURE 2:**
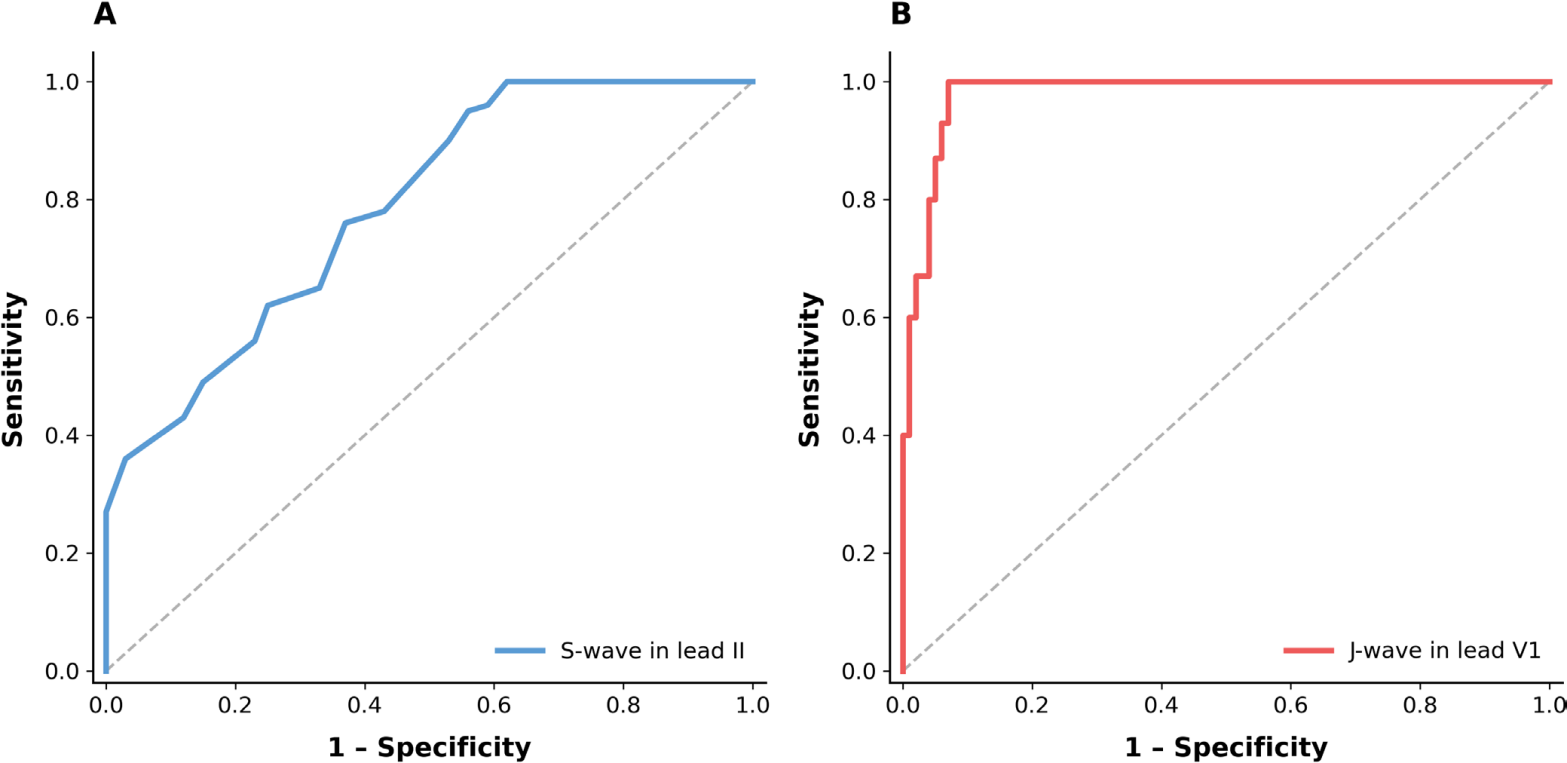
Receiver operating characteristic curve of S‐wave duration in lead II for predicting positive ajmaline challenge (Panel A) and J‐wave amplitude in lead V1 for predicting occurrence of VF events (Panel B).

**FIGURE 3:**
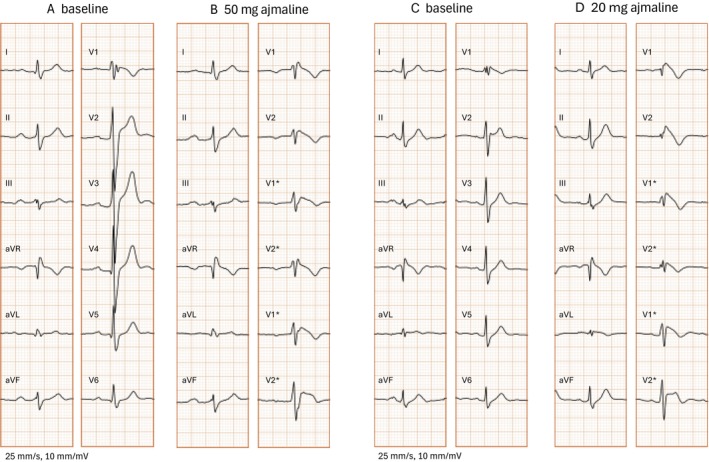
(Panel A–D): Representative examples of baseline ECGs (A and C) without patterns of evident Brugada syndrome. ECGs with prominent deep S‐waves in lead II (panel A) and J‐waves in lead V1 (panel C) are presented. Panel B and D show individual ECG changes upon ajmaline challenge; * marks modified precordial leads recorded during ajmaline challenge. (Panel A, B): 45 y/o male patient diagnosed with Brugada syndrome upon family testing. He was implanted with an ICD after diagnosis due to a pathogenic mutation in the SCN5A gene and inducibility of VF in the electrophysiological study. He remained asymptomatic until age 48 when he developed ventricular fibrillation and appropriate ICD shock. (Panel C, D): 41 y/o male patient with survived cardiac arrest due to ventricular fibrillation. He showed a positive provocation challenge and was diagnosed with Brugada syndrome. During further follow‐up recurrent ICD shocks occurred due to spontaneous episodes of ventricular fibrillation.

Differences of ECG parameters in ajmaline‐positive versus ajmaline‐negative patients are outlined in Table 2 and Table S1. Patients with a positive AC had longer PR durations and wider QRS in the baseline ECG as compared to patients with a negative AC (PR 170 [IQR 35] ms vs. 160 [IQR 30] ms; QRS 96 [IQR 19] ms vs. 90 [IQR 16] ms; *p* = 0.04 and *p* < 0.001, respectively). Type 2 ECG pattern at baseline was more frequent in patients with a positive AC (36% vs. 23%, *p* = 0.048). ROC analysis was performed to identify ECG patterns associated with a higher likelihood of positive test result. Within all tested variables we found that a prominent S‐wave (amplitude and duration) in lead II and J‐wave (amplitude) in lead V1 in the baseline ECG were significantly associated with a positive AC (AUC = 0.76, *p* < 0.001; AUC = 0.79, *p* < 0.001; AUC = 0.71, *p* < 0.001, for S wave amplitude in lead II, S wave duration in lead II and J wave amplitude in lead V1). Sensitivity and specificity of each parameter is shown in Table 3 and the ROC curves are presented in Figure 2A. Youden's index was used to determine the best discriminative cut off value with the highest sensitivity and specificity. Univariate analysis showed a significant correlation between age, occurrence of syncope, PR and QRS duration and positive AC. Further, patients with S‐wave amplitude ≥ 0.1 mV in lead II, S‐wave duration ≥ 19 ms in lead II and J‐wave amplitude ≥ 0.05 mV in lead V1 in the baseline ECG had an increased risk of positive provocation test (OR: 8.9 [CI: 4.1–19.3], OR: 17.3 [CI: 5.9–49.9] and OR: 5.4 [CI: 3.0–10.1]; *p* < 0.001, for S‐wave amplitude and S‐wave duration in lead II and J‐wave amplitude in lead V1) (Table 4). In the multivariable regression analysis J‐wave amplitude ≥ 0.05 mV in lead V1 and S‐wave duration ≥ 19 ms in lead II were independently and significantly associated with a positive AC (OR 5.34 [CI: 2.36–12.06], *p* < 0.001 and OR 9.13 [CI: 2.5–33.52], *p* < 0.001, respectively). When combining these two ECG parameters a positive and negative predictive value of 79% and 73% for predicting a positive AC was achieved. Representative ECGs of prominent S‐waves in lead II and J‐waves in V1 are shown in Figure 3 (panel A, B).

**TABLE 2:** Demographic characteristics and ECG parameters in patients with positive and negative ajmaline challenge.

	Ajmaline positive *N* = 93	Ajmaline negative *N* = 128	*p*
Age, years	42 [IQR 24]	36 [IQR 25]	**0.007**
Male gender, *N* (%)	63 (68%)	94 (73%)	0.37
Body‐mass‐index (kg/m^2^)	25 [IQR 5]	25 [IQR 6]	0.08
Applied dosage of ajmaline (mg/kg body weight)	0.8 [IQR 0.3]	1 [IQR 0.1]	**0.001**
Syncope, *N* (%)	30 (32%)	69 (54%)	**0.002**
Survived sudden cardiac arrest, *N* (%)	15 (16%)	0	n.a.
ECG parameters (quantitative)			
PR duration, ms	170 [IQR 35]	160 [IQR 30]	**0.04**
QRS duration, ms	96 [IQR 19]	90 [IQR 16]	**0.001**
QTc duration, ms	418 [IQR 32]	408 [IQR 33]	0.17
Heart rate, bpm	75 [IQR 20]	70 [IQR 15]	0.05
ECG characteristics (qualitative)			
Presence of first‐degree AV block, *N* (%)	13 (14%)	11 (9%)	0.2
Any right bundle branch block, *N* (%)	56 (60%)	44 (34%)	**0.001**
Complete right bundle branch block, *N* (%)	5 (5%)	1 (1%)	0.08
Type 2 ECG at baseline, *N* (%)	33 (36%)	29 (23%)	**0.04**

**TABLE 3:** Diagnostic performance of different ECG parameters in case of nondiagnostic Brugada pattern.

	Sensitivity (%)	Specificity (%)	AUC	*p*
S‐wave amplitude ≥ 0.1 mV in lead II	90	49	0.76	< 0.001
S‐wave duration ≥ 19 ms in lead II	96	44	0.79	< 0.001
J‐wave amplitude ≥ 0.05 mV in lead V1	56	81	0.71	< 0.001

**TABLE 4:** Clinical and electrocardiographic markers associated with positive test result in the absence of a spontaneous diagnostic type 1 ECG.

	Univariate analysis			Multivariable analysis		
	OR	CI	*p*	OR	CI	*p*
Age	1.03	1.01–1.05	**0.01**	1.03	1.00–1.05	**0.032**
Any type of right bundle branch block	2.9	1.7–5.0	**< 0.001**	1.99	0.88–4.47	0.10
QRS duration	1.04	1.02–1.06	**0.001**	0.99	0.97–1.03	0.82
S‐wave amplitude ≥ 0.1 mV in lead II	8.9	4.1–19.3	**< 0.001**	2.51	0.93–6.8	0.07
S‐wave duration ≥ 19 ms in lead II	17.3	5.9–49.9	**< 0.001**	9.13	2.5–33.52	**< 0.001**
J‐wave amplitude ≥ 0.05 mV in lead V1	5.4	3.0–10.1	**< 0.001**	5.34	2.36–12.06	**< 0.001**
Baseline type 2 ECG	1.9	1.0–3.4	**0.048**	2.4	0.95–6.11	0.07

*Note:* Significance of bold value indicates the statistical significance of *p*‐values when *p* < 0.05.

### 3.4 ECG Markers Associated With History of Ventricular Fibrillation

Baseline ECG characteristics of 15 patients with positive AC and documented VF were compared to baseline ECGs of 78 patients with positive AC, without history of previous VF (Table 5). ROC analysis identified three ECG variables associated with a higher likelihood of VF history: J‐wave amplitude in lead V1 (AUC = 0.98, *p* < 0.001), R‐wave duration in lead aVR (AUC = 0.87, *p* < 0.001), and S‐wave duration in lead I (AUC = 0.78, *p* < 0.001). ROC curve for diagnostic performance of J‐wave amplitude in lead V1 is shown in Figure 2B. Univariate analysis identified J‐wave amplitude in V1 ≥ 0.1 mV, R‐wave duration ≥ 20 ms in lead aVR, and S‐wave duration ≥ 30 ms in lead I to be associated with higher risk of VF (OR: 70 [CI: 8.5–579.9], OR: 1.4 [CI: 1.2–1.7], OR: 14.7 [1.8–117.6], *p* < 0.001 for all parameters). However, in the multivariable analysis only J‐wave in lead V1 was significantly associated with occurrence of VF (Table 6). While the positive predictive value was only 48%, the negative predictive value was 99%. A representative ECG example of a nondiagnostic ECG with history of VF is shown in Figure 3 (panel C, D).

**TABLE 5:** Comparison of demographic and electrocardiographic characteristics of patients with and without a history of ventricular fibrillation.

	Patients with history of VF (*N* = 15)	Patients without history of VF (*N* = 78)	*p*
Age, years	41 (IQR 17)	44 (IQR 22)	0.971
Male gender, *N* (%)	14 (93%)	49 (63%)	**0.032**
Body‐mass‐index (kg/m^2^)	28.8 (IQR 9.4)	24.9 (IQR 4.4)	0.313
Applied ajmaline (mg/kg body weight)	0.78 (IQR 0.16)	0.91 (IQR 0.32)	0.431
Syncope, *N* (%)	1 (7%)	29 (37%)	**0.032**
ECG parameters (quantitative)			
PR duration, ms	164 (IQR 24)	166 (IQR 36)	0.738
QRS duration, ms	104 (IQR 16)	96 (IQR 16)	0.052
QTc duration, ms	419 (IQR 27)	417 (IQR 36)	0.244
ECG characteristics (qualitative)			
Presence of first‐degree AV block, *N* (%)	2 (13%)	11 (14%)	1.0
Complete right bundle branch block, *N* (%)	3 (20%)	2 (3%)	**0.03**
Type 2 ECG at baseline, *N* (%)	6 (40%)	27 (35%)	0.69

*Note:* Significance of bold value indicates the statistical significance of *p*‐values when *p* < 0.05.

**TABLE 6:** Clinical and electrocardiographic markers associated with history of VF in ajmaline‐positive patients.

	Univariate analysis			Multivariable analysis		
	HR	CI	*p*	HR	CI	*p*
Male gender	8.3	1.04–66.33	**0.046**	n.s.	n.s.	n.s.
History of syncope	0.1	0.02–0.97	**0.046**	n.s.	n.s.	n.s.
Complete RBBB	9.5	1.4–62.9	**0.02**	n.s.	n.s.	n.s.
S‐wave duration ≥ 30 ms in lead I	14.7	1.8–117.6	**< 0.001**	n.s.	n.s.	n.s.
R‐wave duration ≥ 20 ms in lead aVR	1.4	1.2–1.7	**< 0.001**	n.s.	n.s.	n.s.
J‐wave amplitude ≥ 0.1 mV in lead V1	70	8.5–579.9	**< 0.001**	41.8	3.1–567.6	**0.005**

*Note:* Significance of bold value indicates the statistical significance of *p*‐values when *p* < 0.05.

## 4 Discussion

We analyzed baseline ECGs of 221 patients who underwent AC for suspicion of BS. ECGs of negative patients were compared to ECGs of positive patients. The main findings are: (1) BS patients with nondiagnostic ECG at baseline have subtle electrocardiographic changes (deep and long S‐waves in lead II) suggesting conduction anomalies, associated with a higher likelihood of a positive AC. (2) Prominent J‐waves in V1 best discriminate AC positive patients with higher risk of ventricular fibrillation in the absence of spontaneous type 1 ECG.

### 4.1 ECG Indicators of Positive AC

One of the main proposed mechanisms in BS is the conduction delay in RVOT (i.e., depolarization theory). The conduction delay in RVOT results in delayed electrical activation of the basal region of the right ventricle/RVOT which leads to an upward, rightward and backward electrical vector at the end of QRS (the third vector) (Bayés de Luna and ABs 2012; Calo et al. 2016). With increasing severity of conduction disturbance in the RVOT, the overall vector in frontal axis would also be pointing to the aVR and moving away from the lead I. However, in patients with nondiagnostic ECG, it would be anticipated to see the subtle conduction delay only at the end of the QRS mirroring the delayed and sole activation of RVOT.

According to our findings, positive AC was more likely in individuals with prominent S‐waves (both in amplitude and duration) in lead II and J‐waves in lead V1. While the individual parameters alone had high test sensitivity, the conclusion was hampered by a lack of specificity and therefore a low positive predictive value. However, when combining the two parameters S‐wave duration in lead II and J‐wave amplitude in lead V1, the positive and negative predictive value could be improved to well above 70%. This finding may be particularly important for screening of relatives in families with unexplained SCD or families with history of BS. To the best of our knowledge, this is the first report of characteristic depolarization patterns associated with a higher risk of positive provocation testing.

Several studies (Veltmann et al. 2009; Tadros et al. 2017) have reported on clinical characteristics and electrocardiographic patterns in patients undergoing AC. Similar to findings of Veltmann et al. (2009)., we found that patients with positive AC had longer PR and QRS durations at baseline. This could indicate to underlying genetic alterations in the depolarizing sodium current *I*_*Na*_ aggravated by pharmacologic sodium channel block (Veeraraghavan and Poelzing 2008). Since genotyping was performed only in the minority of the present study population (2% ajmaline‐negative and 58% ajmaline‐positive) this hypothesis cannot be further explored.

### 4.2 Markers of Increased Risk for Spontaneous Ventricular Tachyarrhythmias

Ventricular fibrillation occurred in 15 patients. In all patients, AC was necessary to confirm the diagnosis of BS. Markers of increased risk for history of VF, in the absence of diagnostic type 1 ECG pattern, were: J‐wave amplitude in V1 ≥ 0.1 mV, R‐wave duration ≥ 20 ms in lead aVR, and S‐wave duration ≥ 30 ms in lead I. In the multivariable logistic regression only J‐wave in V1 ≥ 0.1 mV was independently and significantly associated with ventricular fibrillation. These findings are in line with previously published data (Calo et al. 2016; Babai Bigi et al. 2007; Matsuo et al. 2001).

Numerous studies evaluated ECG findings predictive of spontaneous VF in BS. Unlike other channelopathies in which the disease phenotype correlates to the prognosis (e.g., QT duration in long‐QT syndrome), in BS the severity of the ECG alterations of the type 1 pattern has not a defined role for the prognosis. Furthermore, disease modifying ECG patterns are dynamic and may not always be present (Veltmann et al. 2006). This circadian or even seasonal fluctuation makes risk stratification even more complex and unpredictable. Permanent spontaneous type 1 ECG pattern in precordial or extremity leads is considered the marker of highest prognostic significance (Delise et al. 2018). Other risk factors associated with spontaneous VF events include: fragmented QRS complex (Morita et al. 2018), early repolarization pattern (Kawata et al. 2013), aVR sign (Babai Bigi et al. 2007), and transmural dispersion of repolarization (Tpeak‐Tend interval) (Maury et al. 2015). Tse et al. used automated measurements from raw ECG data and using a weighed score system based on QRS frontal axis, R‐wave duration (lead III), S‐wave duration (lead I), QRS duration (lead I) and ST slope (lead I) created a model that predicted incident spontaneous VT/VF with an AUC of 0.95 (Tse et al. 2020). While manual ECG measurements may be susceptible to variation and errors, this study demonstrated that easily obtainable ECG variables, extracted automatically from raw ECG data, can predict arrhythmic events with good fidelity.

In our study, the absence of significant J‐waves in V1 was highly associated with good clinical outcome. In asymptomatic, low/intermediate risk patients, this parameter could be help to weigh up the implantation of an ICD.

Clinical parameters (i.e., male, age, syncope or type 2 ECG, etc.) were not associated with adverse outcome during follow‐up. However, we recognize the small sample size that limits a general conclusion.

### 4.3 Diagnostic Value and Controversy of Pharmacological Challenge in Brugada Syndrome

Provocation testing with sodium channel blockers (e.g., ajmaline, flecainide, procainamide) is recommended by current guidelines when BS is suspected either due to individual clinical findings (recurrent unexplained syncope, incomplete right bundle branch block, survived cardiac arrest in absence of structural heart disease etc.) or family history (mainly unexplained cardiac arrest in family members at age < 40 years) in the absence of individual symptoms (Zeppenfeld et al. 2022). Patients presenting with spontaneous type 1 ECG should not undergo provocation testing because the additional diagnostic value is limited, and the added prognostic value is not established. In these patients, other conditions that may explain the type 1 pattern (phenocopies) should be excluded.

The specificity and the positive predictive value is considered high (Hong et al. 2004), although false positive results may exist and further complicate the workup (Viskin et al. 2015). This is why today, it seems reasonable to forgo provocation testing in patients with incidental finding of type 2 ECG pattern, without other symptoms (VF/family history) (Wilde et al. 2023). Strikingly, 70% of patients diagnosed with “asymptomatic BS” received this diagnosis after a positive AC (Probst et al. 2010). Today, the most common reason for implantable cardioverter‐defibrillator in asymptomatic patients is a positive pharmacological challenge together with inducible VF in electrophysiological studies (Sacher et al. 2013; Conte et al. 2015). Considering the low rate of appropriate ICD shocks in asymptomatic individuals, particularly in those with drug‐induced type 1 ECG (0.3% per year) (Sroubek et al. 2016), and the high risk of ICD‐related complications, ranging up to 37% (Sacher et al. 2013), it is necessary to critically reappraise the specificity and the meaning of positive sodium channel blocker challenge test (Viskin and Rosso 2017).

In our study, 13/15 patients (87%) with positive AC had history of VF before undergoing the test, and 2 patients with positive AC developed spontaneous VF during follow‐up, after establishing the diagnosis of BS. Both patients were implanted with an ICD based on individual risk stratification. This translates to a 2% risk of spontaneous ventricular tachyarrhythmia after positive AC result during follow‐up (4.9 ± 3.3 years).

### 4.4 Limitations

This is a retrospective study including clinical and ECG data only at the time of AC. Relevant information during follow‐up (e.g., clinical events, ECG changes) was not obtained. The study population was heterogeneous and included patients with documented VF, patients with syncope and minor ECG abnormalities suggestive of BS but also asymptomatic family members of BS patients referred for diagnostic evaluation. Although all ECGs analyzed in the present study were obtained immediately prior to AC, we cannot fully exclude the possibility of dynamic ECG fluctuations, which are known in Brugada patients, that could have biased the result. Finally we acknowledge the small number of patients, especially in the subgroup of patients evaluated for VF risk markers which limits generalization of the results.

## 5 Conclusion

BS patients without spontaneous diagnostic type 1 ECG show subtle but specific electrocardiographic patterns associated with a higher likelihood of positive provocation testing with ajmaline. These findings are particularly relevant for family members of Brugada patients who do not present with a spontaneous diagnostic ECG as they allow triaging of AC in family screening.

## Author Contributions

**Erol Tülümen:** conceptualization, methodology, writing, and formal analysis. **Mathieu Kruska:** investigation, data curation, and validation. **Sara Wuerfel:** formal analysis, investigation, and software. **Maximilian Kohl:** data correction, software, and visualization. **Volker Liebe:** validation, writing, and revision. **Ibrahim Akin:** resources, supervision, and validation. **Juergen Kuschyk:** investigation, methodology, and revision. **Daniel Duerschmied:** supervision, resources, and writing. **Martin Borggrefe:** project administration, supervision, and revision. **Boris Rudic:** supervision, conceptualization, writing, revision, and methodology.

## Conflicts of Interest

The authors declare no conflicts of interest.

## Supporting information


**Table S1:** anec70137‐sup‐0001‐TableS1.docx.

## Data Availability

The data that support the findings of this study are available on request from the corresponding author. The data are not publicly available due to privacy or ethical restrictions.
