# Supplementary material for: ECG Markers of Positive Drug Challenge With Ajmaline in Patients With Brugada Syndrome
Source: Ann Noninvasive Electrocardiol. 2025 Dec 8;31(1):e70137. doi: 10.1111/anec.70137 (PMC12683172; doi:10.1111/anec.70137)
Supplement: Supplementary file 1 — Table S1: anec70137‐sup‐0001‐TableS1.docx. [file ANEC-31-e70137-s001.docx]

**Supplementary**

Table S1: Detailed overview of all analyzed ECG parameters

|  |  | **ECG Measurement** | **Ajmaline-positive**  **N=98** | **Ajmaline-negative**  **N=128** | **P** |
| --- | --- | --- | --- | --- | --- |
|  | ECG lead I | Q-wave duration in lead I (ms) | 9 IQR [18] | 10 IQR [16] | 0.985 |
|  |  | Q-wave amplitude in lead I (mV) | -0.01 IQR [0.04] | -0.02 IQR [0.05] | 0.641 |
|  |  | R-wave duration in lead I (ms) | 44 IQR [23] | 44 IQR [22] | 0.873 |
|  |  | R-wave amplitude in lead I (mV) | 0.74 IQR [0.41] | 0.78 IQR [0.46] | 0.480 |
|  |  | S-wave duration in lead I (ms) | 32 IQR [26] | 26 IQR [36] | **0.015** |
|  |  | S-wave amplitude in lead I (mV) | -0.15 IQR [0.25] | -0.09 IQR [0.19] | **0.020** |
|  |  | J-wave amplitude in lead I (mV) | 0.01 IQR [0.03] | 0.02 IQR [0.04] | **0.022** |
|  |  | ST-segment integral in lead I (mV·ms) | 0.04 IQR [0.04] | 0.04 IQR [0.04] | 0.326 |
|  | ECG lead II | Q-wave duration in lead II (ms) | 0 IQR [14] | 0 IQR [18] | 0.107 |
|  |  | Q-wave amplitude in lead II (mV) | 0 IQR [0.04] | 0 IQR [0.09] | 0.110 |
|  |  | R-wave duration in lead II (ms) | 44 IQR [12] | 45 IQR [13.5] | 0.843 |
|  |  | R-wave amplitude in lead II (mV) | 0.91 IQR [0.5] | 0.82 IQR [0.73] | 0.452 |
|  |  | S-wave duration in lead II (ms) | 36 IQR [20] | 22 IQR [34] | **<0.001** |
|  |  | S-wave amplitude in lead II (mV) | -0.25 IQR [0.2] | -0.11 IQR [0.21] | **<0.001** |
|  |  | J-wave amplitude in lead II (mV) | 0 IQR [0.05] | 0.01 IQR [0.06] | **0.003** |
|  |  | ST-segment integral in lead II (mV·ms) | 0.04 IQR [0.06] | 0.03 IQR [0.07] | 0.221 |
|  | ECG lead III | Q-wave duration in lead III (ms) | 0 IQR [17.5] | 0 IQR [14] | 0.370 |
|  |  | Q-wave amplitude in lead III (mV) | 0 IQR [0.05] | 0 IQR [0.05] | 0.587 |
|  |  | R-wave duration in lead III (ms) | 38 IQR [21.5] | 38 IQR [29.5] | 0.839 |
|  |  | R-wave amplitude in lead III (mV) | 0.38 IQR [0.49] | 0.4 IQR [0.73] | 0.224 |
|  |  | S-wave duration in lead III (ms) | 34 IQR [33.5] | 27 IQR [35.5] | **0.044** |
|  |  | S-wave amplitude in lead III (mV) | -0.16 IQR [0.27] | -0.15 IQR [0.23] | 0.298 |
|  |  | J-wave amplitude in lead III (mV) | -0.01 IQR [0.04] | 0.01 IQR [0.04] | **<0.001** |
|  |  | ST-segment integral in lead III (mV·ms) | 0.01 IQR [0.03] | 0.02 IQR [0.04] | 0.310 |
|  | ECG lead aVR | Q-wave duration in lead aVR (ms) | 35 IQR [51.5] | 0 IQR [44] | 0.010 |
|  |  | Q-wave amplitude in lead aVR (mV) | -0.41 IQR [0.71] | 0 IQR [0.66] | 0.066 |
|  |  | R-wave duration in lead aVR (ms) | 20 IQR [21.5] | 12 IQR [20] | 0.002 |
|  |  | R-wave amplitude in lead aVR (mV) | 0.12 IQR [0.22] | 0.04 IQR [0.11] | **<0.001** |
|  |  | S-wave duration in lead aVR (ms) | 0 IQR [41.5] | 0 IQR [42] | 0.182 |
|  |  | S-wave amplitude in lead aVR (mV) | 0 IQR [0.91] | 0 IQR [0.87] | 0.651 |
|  |  | J-wave amplitude in lead aVR (mV) | 0 IQR [0.04] | 0 IQR [0.02] | **0.001** |
|  |  | ST-segment integral in lead aVR (mV·ms) | -0.03 IQR [0.05] | -0.02 IQR [0.05] | 0.700 |
|  | ECG lead aVL | Q-wave duration in lead aVL (ms) | 10 IQR [20] | 12 IQR [18] | 0.930 |
|  |  | Q-wave amplitude in lead aVL (mV) | -0.015 IQR [0.06] | -0.03 IQR [0.07] | 0.412 |
|  |  | R-wave duration in lead aVL (ms) | 43 IQR [38] | 40 IQR [31] | 0.138 |
|  |  | R-wave amplitude in lead aVL (mV) | 0.35 IQR [0.42] | 0.41 IQR [0.49] | 0.570 |
|  |  | S-wave duration in lead aVL (ms) | 14 IQR [38] | 19 IQR [38] | 0.472 |
|  |  | S-wave amplitude in lead aVL (mV) | -0.06 IQR [0.21] | -0.07 IQR [0.21] | 0.460 |
|  |  | J-wave amplitude in lead aVL (mV) | 0.02 IQR [0.04] | 0.01 IQR [0.03] | **0.025** |
|  |  | ST-segment integral in lead aVL (mV·ms) | 0.02 IQR [0.03] | 0.02 IQR [0.04] | 0.160 |
|  | ECG lead aVF | Q-wave duration in lead aVF (ms) | 0 IQR [14] | 0 IQR [16] | 0.695 |
|  |  | Q-wave amplitude in lead aVF (mV) | 0 IQR [0.04] | 0 IQR [0.05] | 0.648 |
|  |  | R-wave duration in lead aVF (ms) | 44 IQR [16] | 43 IQR [20] | 0.835 |
|  |  | R-wave amplitude in lead aVF (mV) | 0.55 IQR [0.49] | 0.56 IQR [0.59] | 0.917 |
|  |  | S-wave duration in lead aVF (ms) | 36 IQR [23] | 26 IQR [36] | **0.003** |
|  |  | S-wave amplitude in lead aVF (mV) | -0.17 IQR [0.22] | -0.14 IQR [0.24] | **0.049** |
|  |  | J-wave amplitude in lead aVF (mV) | 0 IQR [0.05] | 0.01 IQR [0.04] | **<0.001** |
|  |  | ST-segment integral in lead aVF (mV·ms) | 0.02 IQR [0.04] | 0.03 IQR [0.04] | 0.961 |
|  | ECG lead V1 | Q-wave duration in lead V1 (ms) | 0 IQR [0] | 0 IQR [0] | 0.204 |
|  |  | Q-wave amplitude in lead V1 (mV) | 0 IQR [0] | 0 IQR [0] | 0.247 |
|  |  | R-wave duration in lead V1 (ms) | 22 IQR [9.5] | 22 IQR [8] | 0.151 |
|  |  | R-wave amplitude in lead V1 (mV) | 0.12 IQR [0.12] | 0.16 IQR [0.14] | **0.007** |
|  |  | S-wave duration in lead V1 (ms) | 50 IQR [29.5] | 55 IQR [22] | 0.185 |
|  |  | S-wave amplitude in lead V1 (mV) | -0.67 IQR [0.48] | -0.74 IQR [0.5] | **0.034** |
|  |  | J-wave amplitude in lead V1 (mV) | 0.06 IQR [0.13] | 0.01 IQR [0.04] | **<0.001** |
|  |  | ST-segment integral in lead V1 (mV·ms) | 0.04 IQR [0.08] | 0.03 IQR [0.04] | 0.189 |
|  | ECG lead V2 | Q-wave duration in lead V2 (ms) | 0 IQR [0] | 0 IQR [0] | 0.903 |
|  |  | Q-wave amplitude in lead V2 (mV) | 0 IQR [0] | 0 IQR [0] | 0.908 |
|  |  | R-wave duration in lead V2 (ms) | 31 IQR [17.5] | 30 IQR [14] | 0.400 |
|  |  | R-wave amplitude in lead V2 (mV) | 0.37 IQR [0.42] | 0.45 IQR [0.5] | 0.086 |
|  |  | S-wave duration in lead V2 (ms) | 52 IQR [22] | 50 IQR [16] | 0.236 |
|  |  | S-wave amplitude in lead V2 (mV) | -0.82 IQR [0.57] | -1 IQR [0.78] | **0.014** |
|  |  | J-wave amplitude in lead V2 (mV) | 0.07 IQR [0.15] | 0.05 IQR [0.09] | 0.088 |
|  |  | ST-segment integral in lead V2 (mV·ms) | 0.09 IQR [0.13] | 0.11 IQR [0.14] | 0.160 |
|  | ECG lead V3 | Q-wave duration in lead V3 (ms) | 0 IQR [0] | 0 IQR [0] | 0.210 |
|  |  | Q-wave amplitude in lead V3 (mV) | 0 IQR [0] | 0 IQR [0] | 0.214 |
|  |  | R-wave duration in lead V3 (ms) | 43 IQR [10] | 42 IQR [10] | 0.445 |
|  |  | R-wave amplitude in lead V3 (mV) | 0.7 IQR [0.68] | 0.92 IQR [0.73] | **0.007** |
|  |  | S-wave duration in lead V3 (ms) | 48 IQR [20] | 40 IQR [20] | **<0.001** |
|  |  | S-wave amplitude in lead V3 (mV) | -0.7 IQR [0.64] | -0.6 IQR [0.48] | 0.173 |
|  |  | J-wave amplitude in lead V3 (mV) | 0.01 IQR [0.06] | 0.03 IQR [0.07] | **<0.001** |
|  |  | ST-segment integral in lead V3 (mV·ms) | 0.09 IQR [0.1] | 0.1 IQR [0.08] | 0.216 |
|  | ECG lead V4 | Q-wave duration in lead V4 (ms) | 0 IQR [4.5] | 0 IQR [12] | 0.145 |
|  |  | Q-wave amplitude in lead V4 (mV) | 0 IQR [0.02] | 0 IQR [0.02] | 0.255 |
|  |  | R-wave duration in lead V4 (ms) | 44 IQR [8] | 43 IQR [8] | 0.602 |
|  |  | R-wave amplitude in lead V4 (mV) | 1.16 IQR [0.71] | 1.8 IQR [0.65] | 0.127 |
|  |  | S-wave duration in lead V4 (ms) | 42 IQR [19.5] | 32 IQR [23.5] | **<0.001** |
|  |  | S-wave amplitude in lead V4 (mV) | -0.41 IQR [0.43] | -0.31 IQR [0.36] | 0.013 |
|  |  | J-wave amplitude in lead V4 (mV) | 0 IQR [0.05] | 0.03 IQR [0.06] | **<0.001** |
|  |  | ST-segment integral in lead V4 (mV·ms) | 0.06 IQR [0.08] | 0.06 IQR [0.06] | 0.289 |
|  | ECG lead V5 | Q-wave duration in lead V5 (ms) | 0 IQR [16] | 10 IQR [16] | 0.356 |
|  |  | Q-wave amplitude in lead V5 (mV) | 0 IQR [0.05] | -0.02 IQR [0.06] | 0.275 |
|  |  | R-wave duration in lead V5 (ms) | 44 IQR [8] | 42 IQR [10] | 0.635 |
|  |  | R-wave amplitude in lead V5 (mV) | 1.06 IQR [0.49] | 1.24 IQR [0.45] | **0.007** |
|  |  | S-wave duration in lead V5 (ms) | 39 IQR [20] | 30 IQR [27.5] | **<0.001** |
|  |  | S-wave amplitude in lead V5 (mV) | -0.22 IQR [0.28] | -0.16 IQR [0.22] | **0.007** |
|  |  | J-wave amplitude in lead V5 (mV) | 0 IQR [0.03] | 0.02 IQR [0.04] | **<0.001** |
|  |  | ST-segment integral in lead V5 (mV·ms) | 0.04 IQR [0.06] | 0.04 IQR [0.05] | 0.261 |
|  | ECG lead V6 | Q-wave duration in lead V6 (ms) | 12 IQR [18] | 14 IQR [18] | 0.156 |
|  |  | Q-wave amplitude in lead V6 (mV) | -0.02 IQR [0.06] | -0.03 IQR [0.07] | 0.067 |
|  |  | R-wave duration in lead V6 (ms) | 48 IQR [13.5] | 46 IQR [20] | 0.541 |
|  |  | R-wave amplitude in lead V6 (mV) | 0.84 IQR [0.31] | 1.01 IQR [0.38] | **<0.001** |
|  |  | S-wave duration in lead V6 (ms) | 34 IQR [32] | 26 IQR [38] | **0.009** |
|  |  | S-wave amplitude in lead V6 (mV) | -0.11 IQR [0.18] | -0.06 IQR [0.15] | **0.036** |
|  |  | J-wave amplitude in lead V6 (mV) | 0 IQR [0.02] | 0.02 IQR [0.04] | **<0.001** |
|  |  | ST-segment integral in lead V6 (mV·ms) | 0.03 IQR [0.04] | 0.03 IQR [0.04] | 0.161 |
